# PGRP-LD mediates *A*. *stephensi* vector competency by regulating homeostasis of microbiota-induced peritrophic matrix synthesis

**DOI:** 10.1371/journal.ppat.1006899

**Published:** 2018-02-28

**Authors:** Xiumei Song, Mengfei Wang, Li Dong, Huaimin Zhu, Jingwen Wang

**Affiliations:** 1 State Key Laboratory of Genetic Engineering, School of Life Sciences, Fudan University, Shanghai, P. R. China; 2 Ministry of Education Key Laboratory of Contemporary Anthropology, School of Life Sciences, Fudan University, Shanghai, P. R. China; 3 The 2nd Military Medical University, Shanghai, P. R. China; Johns Hopkins University, Bloomberg School of Public Health, UNITED STATES

## Abstract

Peptidoglycan recognition proteins (PGRPs) and commensal microbes mediate pathogen infection outcomes in insect disease vectors. Although PGRP-LD is retained in multiple vectors, its role in host defense remains elusive. Here we report that *Anopheles stephensi* PGRP-LD protects the vector from malaria parasite infection by regulating gut homeostasis. Specifically, knock down of PGRP-LD (dsLD) increased susceptibility to *Plasmodium berghei* infection, decreased the abundance of gut microbiota and changed their spatial distribution. This outcome resulted from a change in the structural integrity of the peritrophic matrix (PM), which is a chitinous and proteinaceous barrier that lines the midgut lumen. Reduction of microbiota in dsLD mosquitoes due to the upregulation of immune effectors led to dysregulation of PM genes and PM fragmentation. Elimination of gut microbiota in antibiotic treated mosquitoes (Abx) led to PM loss and increased vectorial competence. Recolonization of Abx mosquitoes with indigenous *Enterobacter sp*. restored PM integrity and decreased mosquito vectorial capacity. Silencing PGRP-LD in mosquitoes without PM didn’t influence their vector competence. Our results indicate that PGPR-LD protects the gut microbiota by preventing hyper-immunity, which in turn promotes PM structurally integrity. The intact PM plays a key role in limiting *P*. *berghei* infection.

## Author summary

Malaria parasites must overcome several obstacles to complete their development in mosquito. Understanding the interactions between parasites and mosquitoes will provide potential targets to control malaria transmission. PGRP-LD is a peptidoglycan recognition protein, of which limit information is available in insects. Here we show that *A*. *stephensi* PGRP-LD mediates malaria parasite infection outcomes by influencing homeostasis of the gut microbiota. Reduction of the gut microbiota density, resulting from upregulation of immune activities in PGRP-LD knock down mosquitoes, changes expression of PM genes and causes PM fragmentation. The compromised PM leads to increasing susceptibility to parasite infection. We also discovered that the PM is lost in mosquitoes in which the gut microbiota is removed by antibiotic treatment. Knock down of PGRP-LD in these mosquitoes doesn’t increase their vector competence. Altogether, these results indicate that capacity of *Anopheles* mosquito to transmit parasites is determined by a finely tuned balance between host immunity, gut microbiota and peritrophic matrix. PGRP-LD is a key mediator in regulating this balance. Our results expand knowledge on interactions between immune system, gut microbiota and *Plasmodium*, and will shed light on equivalent processes in other disease transmitting vectors.

## Introduction

Malaria is caused by parasites from the genus *Plasmodium*. The disease kills over 500,000 people annually, most of which are children under the age of 5 [1]. In order to transmit between humans, *Plasmodium* must overcome several obstacles to complete its development in *Anopheles* mosquitoes [2–4]. The peritrophic matrix (PM), and immuno-competent midgut epithelial cells, are two barriers that interfere with parasite transmission through their mosquito vector. The PM is non-cellular and composed of chitin fibrils and chitin-binding proteins. The structure lines the midgut lumen and wraps the food bolus within the endoperitrophic space, thus protecting the epithelium from abrasive food particles and enteric pathogens [5]. The tight junctions between midgut epithelial cells form another contiguous barrier against parasite invasion [6]. Midgut epithelial cells invaded by *Plasmodium* undergo apoptosis and are replaced by new cells. This rapid turnover not only maintains the integrity of the epithelium, but also clears invading parasites [7]. In addition to overcoming physical barriers present in the mosquito midgut, epithelial cells in this environment also present robust cellular and humoral immunity [3]. This activity includes the synthesis of antimicrobial peptides (AMPs), reactive oxygen species (ROS) and nitric oxide (NO), all of which contribute to parasite clearance [4]. *Anopheles* mosquitoes have 3 types of hemocytes: granulocytes, oenocytoids and prohemocytes [3]. These cells eliminate pathogens via phagocytosis and encapsulation. Hemocytes are also important in *Plasmodium*-mediated immune memory, which enhances the mosquito’s ability to clear parasites upon reinfection [8]. In addition, complement like protein TEP1 by forming TEP1/LRIM1/APL1C complex, is another key systemic antiplasmodial immune mechanism that recognizes and eliminates *Plasmodium* ookinetes in the midgut [3]. Three major immune signaling pathways, Toll, IMD (Immune Deficiency) and JAK/STAT, are critical mediators of malaria infection dynamics in *Anopheles* mosquitoes [3].

Peptidoglycan recognition proteins (PGRP) are pattern recognition molecules that function as receptors and regulators of the Toll and IMD signaling pathways [9]. *Anopheles* has 7 *PGRP* genes, 4 in the Long subfamily (including *PGRP-LA*, *-LB*, *-LC* and *-LD*) and 3 in the short subfamily (*PGRP-S1*, *-S2* and –*S3*) [10]. *Anopheles* PGRP-LC is a receptor of the Immune Deficiency (Imd) pathway that is responsible for triggering synthesis of downstream effector molecules [11]. Knock down of PGRP-LC results in increasing susceptibility to *Plasmodium* infection. PGRP-LA, another receptor of the Imd pathway, protects *A*. *coluzzii* from *Plasmodium* infection in a manner similar to that of PGRP-LC [12]. PGRP-LB, a negative regulator of the Imd pathway, has a dual role in *Anopheles* mosquitoes, facilitating parasite infection and protecting natural gut bacteria [12,13]. However, mechanisms of other PGRPs in response to parasite infection are still inadequate.

The gut microbiota is another important factor that strongly influences vector competence [14]. Interactions between enteric bacteria and the mosquito immune system help to maintain gut homeostasis and protect mosquitoes from pathogens infection [13,15–17]. In the absence of gut microbes, *Anopheles* become highly susceptible to *Plasmodium* infection. Co-feeding parasites with bacteria restores resistance to parasite infection in mosquitoes previously treated with antibiotics to remove their indigenous microbiota. Gut microbes also induce expression of several immune molecules, including antimicrobial peptides and pattern recognition receptors [13], and enhance vector refractoriness by promoting hemocyte differentiation [8]. Some residential bacteria, including *Enterobacter* and *Chromobacterium* isolated from field mosquitoes, directly inhibit parasite infection by secreting secondary metabolites such as reactive oxygen species [15,18].

In this study, we examined the function of PGRP-LD in *A*. *stephensi* and found that this receptor protects the mosquito against *Plasmodium* infection. PGRP-LD helped maintain homeostasis of the mosquito gut microbiota by negatively regulating innate immune responses. The healthy microbiota in turn contributed to the integrity of PM, and the intact PM enhanced *Anopheles* resistance to malaria parasites. Our results suggest that a finely tuned balance between the immune system, gut microbes and the PM is key to determining the capacity of mosquitoes to transmit malaria.

## Results

### PGRP-LD helps to defend against parasite infection

The putative *Anopheles stephensi* PGRP-LD is 42 kD transmembrane protein with 77% identity to *Anopheles gambiae* PGRP-LD. Sequence analysis indicates that it has a peptidoglycan-binding domain. However, the putative protein lacks most of the residues essential for PGN binding and catalytic activity, which are well characterized domains of *Drosophila* PGRPs (S1 Fig).

**Fig 1:**
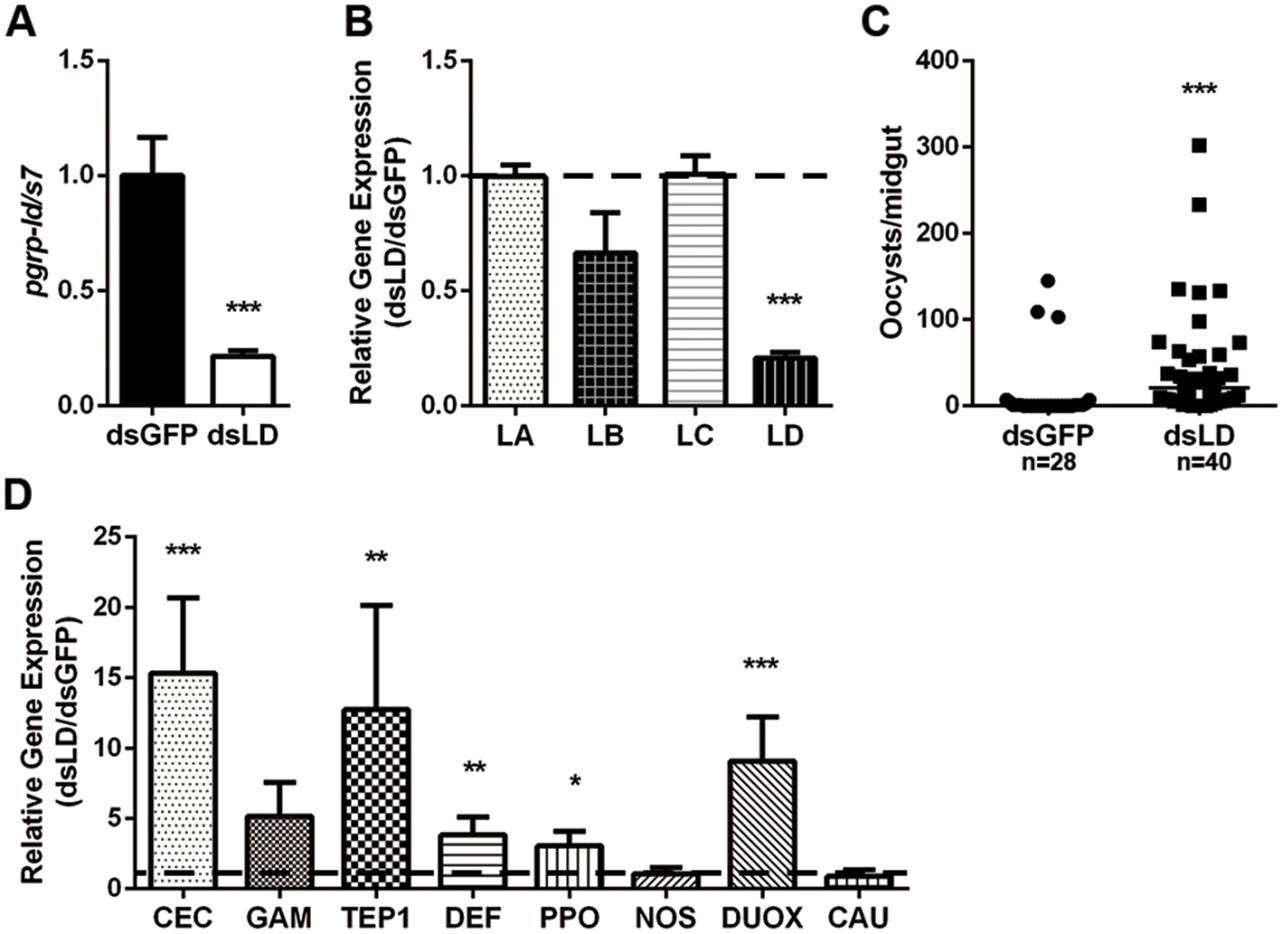
Influence of PGRP-D on *P*. *berghei* infection in *A*. *stephensi*. (A) *pgrp-ld* silencing efficiency and specificity (B) in mosquitoes. Expression level of *pgrp-ld* was normalized to *A*. *stephensi s7*. Relative expression level of *pgrp-ld* in dsLD mosquitoes was normalized to the gene’s expression in dsGFP controls. (C) Oocyst number in dsRNA treated mosquitoes. Median oocysts number is indicated by the horizontal black bar. Each dot represents an individual mosquito. (D) Relative gene expression levels in dsRNA treated mosquitoes 26hr post parasite infection. (A, B and D) Error bars indicate standard error (*n* = 10). Significance was determined by Student’s-T test in (A), (B) and (D), by Mann-Whitney test in (C). *, P<0.05, **, P<0.01, ***, P<0.001. Results from one of three independent experiments are shown.

To investigate the role of PGRP-LD in parasites defense, we knocked down its expression *in vivo* via microinjection of gene-specific double stranded RNA and then analyzed the susceptibility of treated mosquitoes to infection with *P*. *berghei*. The level of *pgrp-ld* was reduced by approximately 67% 2-days post dsRNA treatment compared to dsGFP controls (Fig 1A), and we observed no significant cross reactivity with other long PGRPs, including PGRP-LA, -LB, -LC (Fig 1B). Knock down of *pgrp-ld* didn’t influence the survival rate of mosquitoes (S2 Fig). However, reduced PGRP-LD (dsLD) resulted in a significant increase in the number of oocysts from 0 in dsGFP to 31 in dsLD mosquitoes (Fig 1C).

As PGRPs play important roles in activation and regulation of immune responses, we hypothesized that increased susceptibility of dsLD mosquitoes to parasites infection might resulted from the dysregulation of innate immune responses [9]. To address this question we next analyzed expression of 8 immune genes in dsLD and dsGFP treated mosquitoes 26hr post parasite challenge. The genes we investigated encoded 3 antimicrobial peptides (Cecropin, Gambicin and Defensin), 1 negative regulator of IMD signaling pathway (Caudal) and 4 proteins related to cellular and epithelial immune responses (TEP1, PPO, NOS and DUOX) [3,19]. Interestingly, most of the effector encoding genes, including *cecropin*, *defensin*, *tep1*, *ppo* and *duox*, were significantly upregulated in response to parasite challenge (Fig 1D). However, these induced effectors did not control parasite infection outcomes. This finding suggests a discrepancy exists between increased susceptibility to parasites and enhanced expression of immune genes in the absence of PGRP-LD.

### PGRP-LD contributes to the homeostasis of gut microbiota

**Fig 2:**
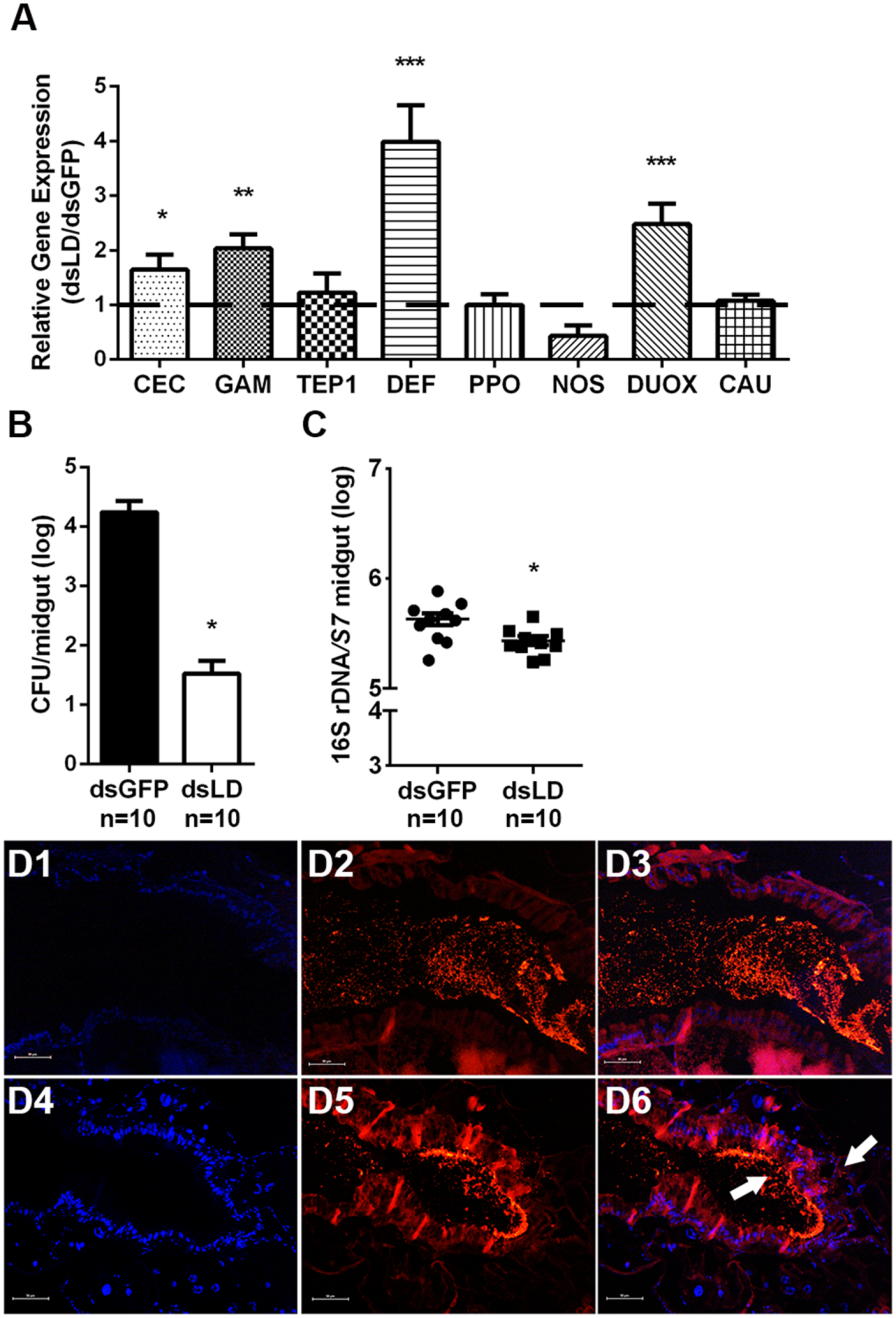
Influence of PGRP-LD on the gut microbiota. (A) Relative expression levels of immunity-related genes in dsRNA treated, sugar fed mosquitoes. Error bars indicate standard error (n = 10). Culturable (B) and total gut microbiota (C) density was measured. Error bars indicate standard error (n = 10). Significance was determined by Student’s-T test. *, P<0.05, **, P<0.01, ***, P<0.001. Results from one of two independent experiments are shown. (D) Microbiota localization in midguts of dsGFP (D1-D3) and dsLD (D4-D6) were analyzed by FISH using a universal bacterial 16S rRNA gene probe (red). Nuclei were stained with DAPI (blue). D1 and D4 show DAPI staining. D2 and D5 show staining with 16S rRNA probe. D3 and D6 show merged images. Arrows denote gut microbiota. Images are representative of three independent experiments. Scale bars, 50 μm.

We next examined if *pgrp-ld* similarly regulated immune responses in mosquitoes prior to blood meal. The same 8 genes were expressed in mosquitoes fed only on sugar. As expected, 4 of these genes (*cecropin*, *gambicin and defensin*, and *duox*) were upregulated in dsLD treated mosquitoes, while *tep1*, *ppo*, *nos* and *caudal* expression remained unchanged (Fig 2A). As both antimicrobial peptides and ROS present bactericidal activities, we next examined if over-activated immune responses exerted an influence on microbiota homeostasis [20,21]. Bacterial load of both culturable and unculturable bacteria were measured in dsLD mosquitoes before consumption of a blood meal. In agreement with our hypothesis, knock down of *pgrp-ld* resulted in an ~500 times reduction of culturable microbes such that dsGFP individuals housed average 1.7X10^4^ CFU/midgut, while dsLD individuals housed average 3.3X10^1^ CFU/midgut (Fig 2B). Similarly, the 16s rRNA gene copy number was significantly lower in dsLD compared to dsGFP mosquitoes (Fig 2C). We next analyzed if community structure of the gut microbiota was influenced in the absence of PGRP-LD. Midguts of dsRNA treated mosquitoes were dissected and bacterial community structure was determined by 16S rRNA next generation sequencing. No significant difference in taxonomic structure was observed between microbial communities in dsGFP and dsLD mosquitoes (S3 Fig). These results indicate that over-activated immune responses in the presence of reduced *pgrp-ld* expression leads to a reduction in the number of residential bacteria, without influencing the taxonomic composition of the gut microbial community. Thus, PGRP-LD helps to protect commensal bacteria by preventing the overactivation of host immune responses.

In addition to investigating bacterial abundance and taxonomic composition, we also examined the spatial distribution of residential bacteria in dsLD mosquito midguts. Localization of residential bacteria in *A*. *stephensi* midguts was examined 48hr post blood meal, which is when cumulative population reaches its maximum density as determined by fluorescent in situ hybridization (FISH) using a universal 16s ribosomal RNA (rRNA) gene probe [22]. We observed a clear physical separation of gut microbiota and epithelium in dsGFP controls (Fig 2D1–2D3). However, dsLD treated mosquitoes exhibited a defect in spatial segregation, with increasing bacteria coming into direct contact with the gut epithelium, and even penetrating epithelial cells (Fig 2D4–2D6). Taken together, these results suggest that PGRP-LD helps to maintain the spatial homeostasis of gut microbes.

### Peritrophic matrix is compromised in the absence of PGRP-LD

**Fig 3:**
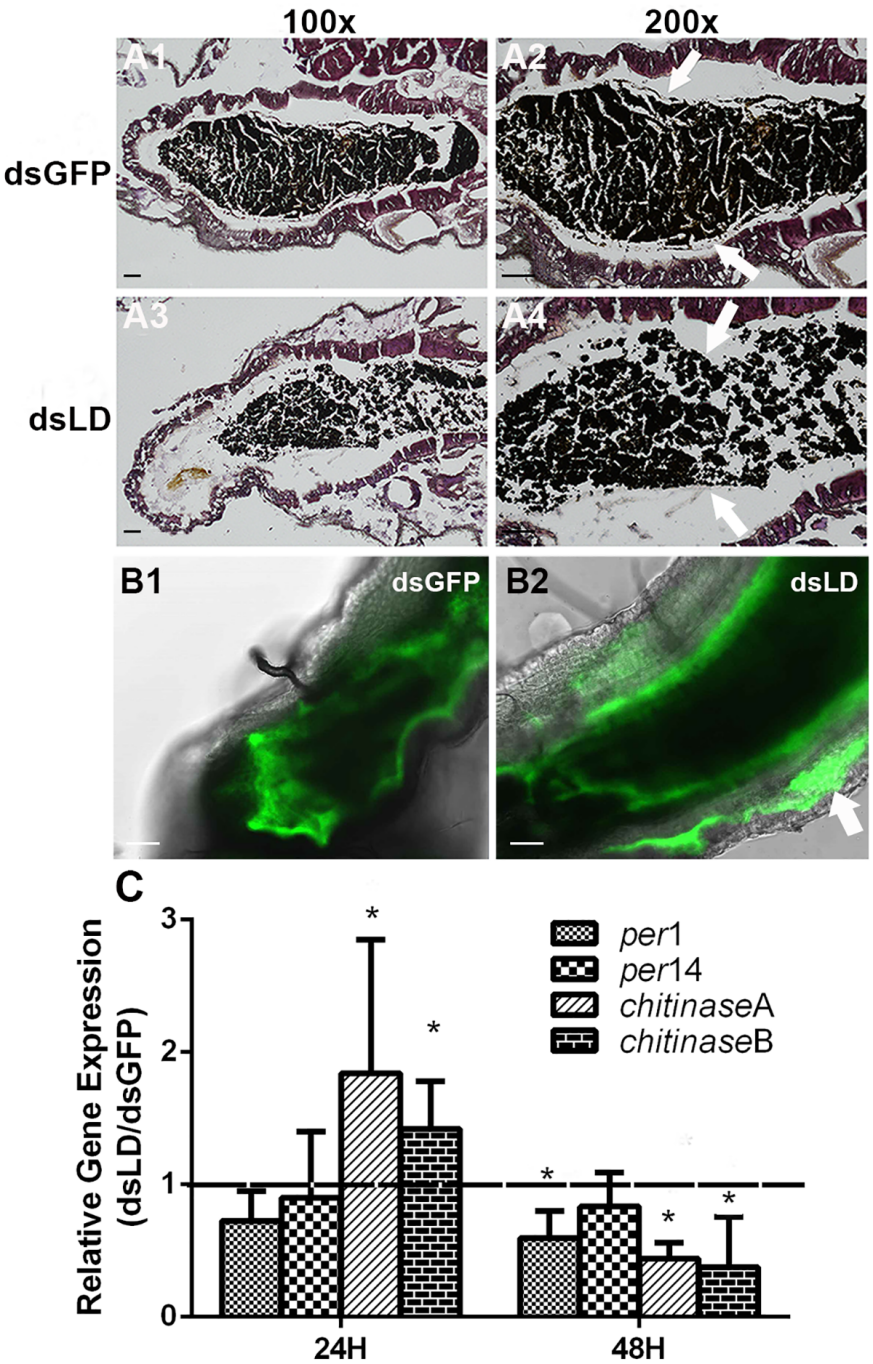
PM structure in dsRNA treated mosquitoes. (A) PM structure was observed by H&E staining in dsGFP (A1 and A2) and dsLD (A3 and A4) mosquitoes at 100X (A1 and A3) and 200X (A2 and A4) magnification. Arrows denote the PM. Scale bars, 50 μm. (B) Dextran-feeding assay in dsRNA treated mosquitoes. The FITC signal is retained in the lumen of dsGFP control mosquitoes, which indicates that the dextran beads are contained within the PM (B1). The FITC signal is observed within gut epithelial cells (indicated by arrow) in dsLD mosquitoes, indicating that the beads can cross the PM (B2). Scale bars, 50 μm. Images are representative of at least two independent experiments. (C) PM gene expression in dsRNA treated mosquitoes. Relative gene expression level in dsRNA treated mosquitoes 24hr and 48 hr post blood meal. Error bars indicate standard error (n = 10).

The PM, which is composed of chitin fibrils and glycoproteins, is a sheath like structure that lines the digestive tract of most insect midguts and prevents luminal contents from coming into direct contact with midgut epithelial cells [23,24]. Mosquitoes have type I PMs, the formation of which is triggered by ingestion of a blood meal [5]. We hypothesized that the microbial diffusion we observed in dsLD midguts may occur because these mosquitoes present a structurally compromised PM. We thus analyzed PM structure in dsLD and dsGFP mosquitoes by hematoxylin and eosin (H&E) and Periodic Acid Schiff (PAS) staining. A fully formed PM was visualized in dsGFP controls 48 hr post blood meal (Figs 3A1 and 3A2 and S4A1). Conversely, the PM of dsLD mosquitoes appeared fragmented (Figs 3A3 and 3A4 and S4A2). To further confirm the impaired PM structure in dsLD mosquitoes, dsRNA treated individuals were fed a blood meal supplemented with FITC-labelled dextran molecules (500 kDa). We observed dextran beads were restrained within the endoperitrophic space in dsGFP mosquitoes 48 hr post feeding (Fig 3B1). In contrast, we observed beads penetrating gut epithelial cells in dsLD mosquitoes, indicating that PM structure was compromised when *pgrp-ld* expression was experimentally reduced (Fig 3B2). We next examined if impaired PM structure was due to the dys-regulation of PM genes. We monitored expression of 2 *peritrophin* genes (*peritrophin1* and *14*), and 2 *chitinases (chitinaseA* and *chitinaseB)*, all of which are involved in the PM formation and degeneration, in dsRNA treated mosquitoes 24hr and 48hr post blood feeding [25,26]. When *pgrp-ld* expression was knocked down, the 2 *chitinases* were upregulated 24 hr post blood meal, followed by a significant downregulation 48hr post blood meal comparing to dsGFP controls. Expression of *peritophin 1* was lower at both time points, with a significant reduction 48hr post blood meal (Fig 3C). These data reinforce our hypothesis that the compromised PM in dsLD mosquitoes is due to the dysregulation of PM associated genes. Taken together, these results suggest that PGRP-LD plays a role in maintaining PM structural integrity in the gut of *A*. *stephensi*.

### Gut microbiota promotes structural integrity of peritrophic matrix

**Fig 4:**
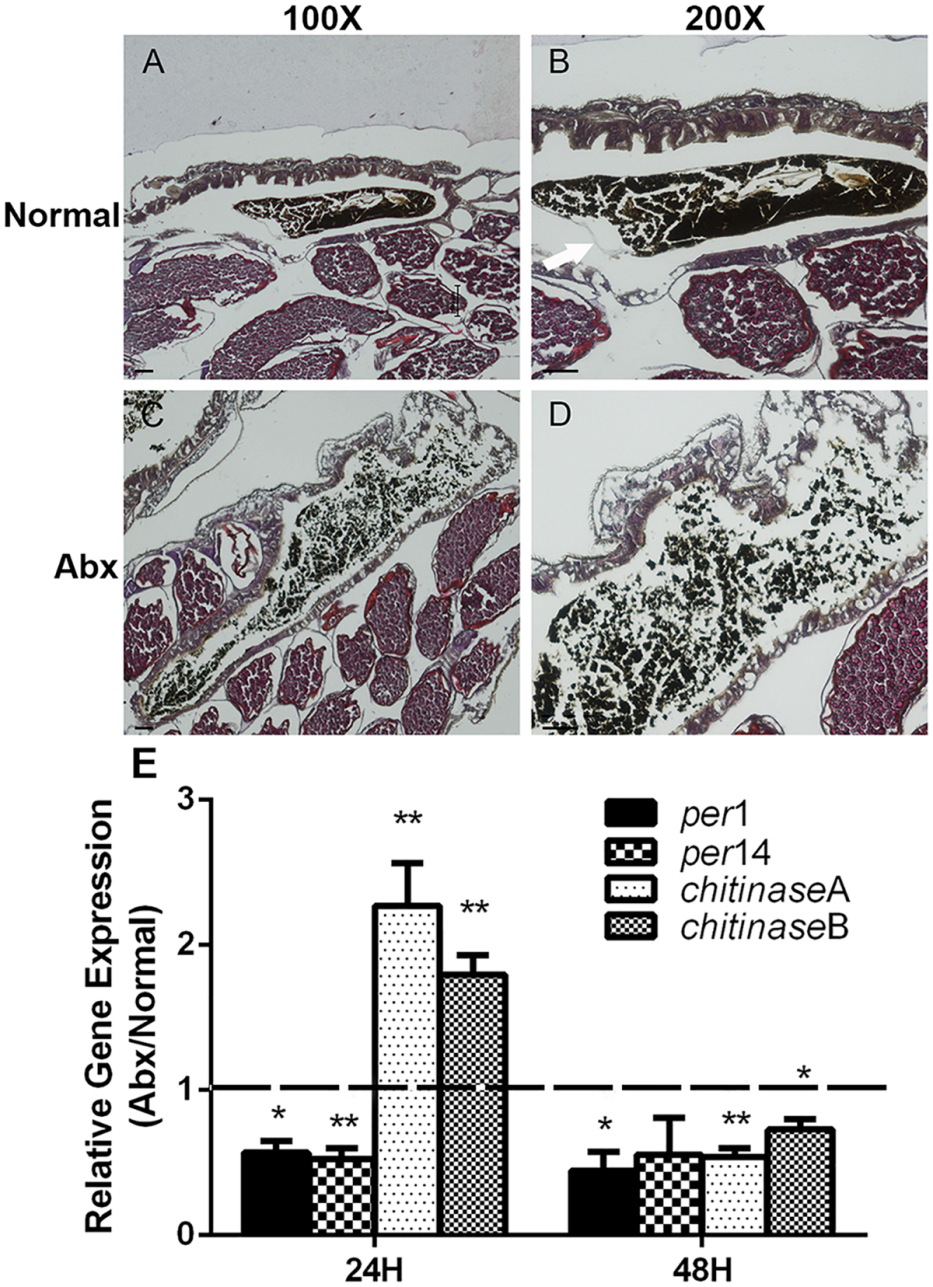
PM structural integrity in normal and antibiotic treated mosquitoes. PM structure was observed by H&E staining in normal (A and B) and antibiotic treated mosquitoes (C and D) at 100X (A and C) and 200X (B and D) magnification. Arrows denote the PM. Images are representative of three independent experiments. Scale bars, 50 μm. (E) Quantification of PM related gene expression in normal and antibiotic treated mosquitoes 24hr and 48 hr post blood meal. Error bars indicate standard error (n = 10).

Gut microbes promote PM structural integrity [27–29]. Because gut microbe abundance was significantly reduced in dsLD mosquitoes, impaired PM structure in these mosquitoes may be due to gut dysbiosis. We next analyzed if resident microbes impact PM structure in *A*. *stephensi*. We again examined the structure of the PM in both normal and antibiotic treated mosquitoes (Abx) 48-hour post blood meal by H&E staining. Antibiotic treatment cleared the majority of native gut bacteria (S5 Fig). Furthermore, an intact PM was observed in guts of normal mosquitoes, which contained the blood bolus within the endoperitrophic space (Fig 4A and 4B). In contrast, when the gut microbiota was removed, no PM was observed and blood was dispersed within the entire gut lumen (Fig 4C and 4D). We again analyzed expression of the same 4 PM genes and found similar expression profiles as in dsRNA treated mosquitoes, with a decrease in the expression of *peritrophin1* and *14* and an initial increase of PM digesting *chitinases* 24 hr post blood meal in antibiotic treated mosquitoes (Fig 4E). Thus, gut microbes may play a role in regulating expression of PM genes, thereby maintaining PM structural integrity.

**Fig 5:**
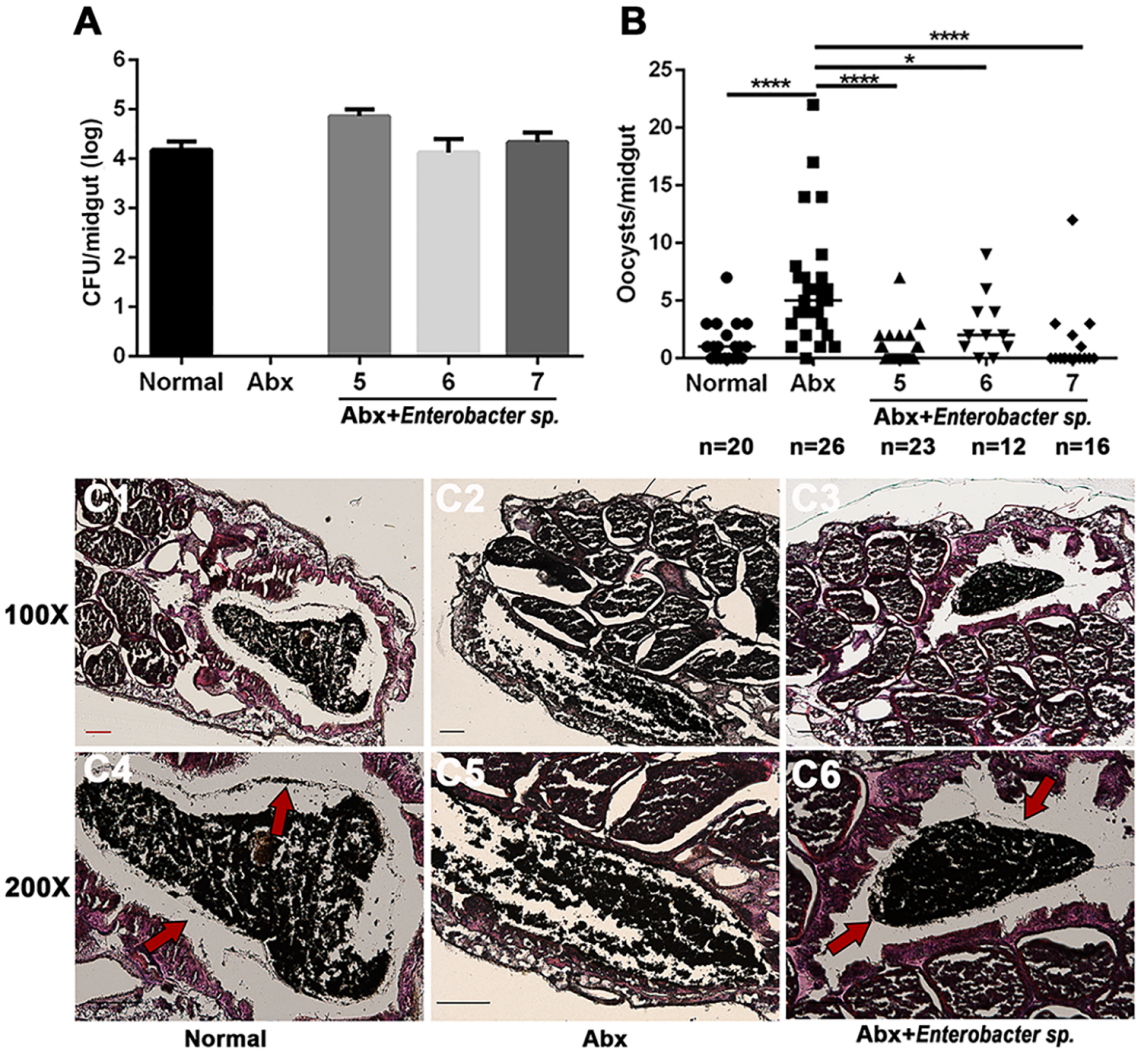
The gut microbiota promotes PM formation. (A) Culturable gut microbiota density in normal, antibiotic treated (Abx) and antibiotic treated mosquitoes recolonized with 1X10^5^/ml (5), 1X10^6^/ml (6) and 1X10^7^/ml (7) *Enterobacter sp*. (B) Infection rate of *P*. *berghei* in normal, antibiotic treated (Abx) and antibiotic treated mosquitoes recolonized with 1X10^5^/ml (5), 1X10^6^/ml (6) and 1X10^7^/ml (7) of *Enterobacter sp*. Median oocyst number is indicated by horizontal black bars. Each dot represents an individual mosquito. (C) PM structure was stained by H&E in normal (C1 and C4), antibiotic treated (C2 and C5) and antibiotic treated mosquitoes recolonized with 1X10^5^/ml *Enterobacter sp*. (C3 and C6) with 100X (C1-3) and 200X (C4-6) magnification. Arrows denote the PM. Images are representative of two independent experiments. Scale bars, 100 μm.

To further analyze the functional association between gut microbes and PM structure, we colonized guts of antibiotic treated mosquitoes with *Enterobacter sp*. (three different doses, 1X10^5^/ml, 10^6^/ml and 10^7^/ml 1.5% sugar solution) prior to administering a blood meal. As *Enterobacter cloacae* is able to inhibit *Plasmodium* infection in *A*. *stephensi* [30], we then examined if *Enterobacter sp*. isolated from our mosquito colony were able to inhibit parasite colonization. Two days post-inoculation, each concentration reached an average density of 7.2X10^4^/ midgut, 1.3X10^4^/midgut and 2.2X10^4^/midgut, respectively, which is comparable to that found indigenously in normal mosquitoes (1.5X10^4^ CFU/midgut) (Fig 5A). We next examined the infection rate in these mosquitoes and found that increasing susceptibility to *P*. *berghei* infection was rescued to normal levels when Abx treated mosquitoes were re-colonized with all three *Enterobacter* concentrations (Fig 5B). Because no difference in infection rate was observed in the three inoculation concentrations, the PM of mosquitoes recolonized with 1X10^5^/ml *Enterobacter sp*. was stained with H&E and PAS 2-day post blood meal. Clear PM structures were observed in both normal mosquitoes (Figs 5C1 and 5C4 and S4B1) and mosquitoes supplemented with *Enterobacter sp*. (Figs 5C3 and 5C6 and S4B3). Conversely, no PM was observed in antibiotic treated individuals (Figs 5C2 and 5C5 and S4B2). These results suggest that the presence of gut microbes is essential to maintain the structural integrity of the PM during blood feeding.

### PM influences outcomes of *Plasmodium* infection

**Fig 6:**
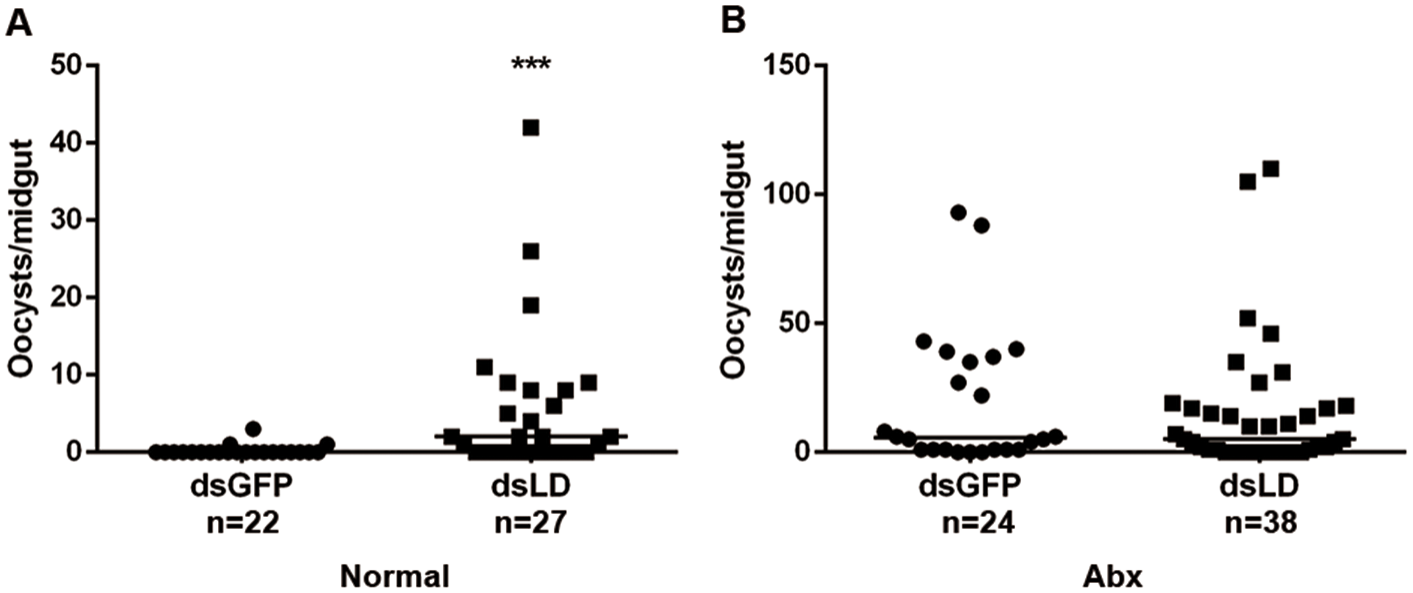
Influence of PGRP-LD on parasitism in normal and antibiotic treated mosquitoes. (A) Oocyst numbers in normal mosquitoes treated with dsRNA. (B) Oocyst numbers in antibiotic treated mosquitoes that received dsRNA. Median oocyst number is indicated by horizontal black bars. Each dot represents an individual mosquito. Significance was determined by Mann-Whitney test. ***, P<0.001. Results from one of three independent experiments are shown.

The PM functions as a physical barrier in mosquito that limits *Plasmodium* infection [31,32]. To further analyze whether the increasing susceptibility in dsLD mosquitoes was due to a compromised PM, we next silenced PGRP-LD in antibiotic treated mosquitoes that lacked a PM and then monitored their susceptibility to parasite infection. In agreement with our previous results, silencing PGRP-LD led to an 8 fold increase in oocyst numbers in dsLD mosquitoes comparing to dsGFP controls (Fig 6A). However, no detectable difference of oocysts number was observed in antibiotic treated mosquitoes injected with dsRNAs (Fig 6B). This result further confirms that enhanced susceptibility to *Plasmodium* infection in dsLD mosquitoes results from the comprised PM. Together, these results indicate that PGRP-LD helps to maintain homeostasis of the gut microbiota by negatively regulating immune responses. The healthy gut microbes promotes the structural integrity of PM. The intact PM functions as a physical barrier that reduces the capacity of parasites to establish infection in mosquitoes.

## Discussion

**Fig 7:**
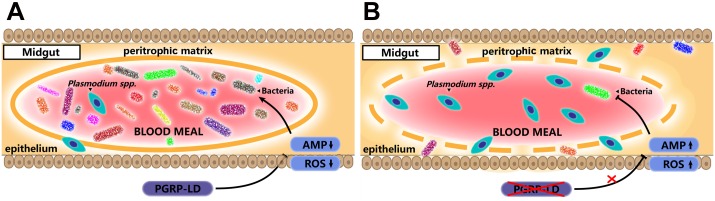
Model of influence of PGRP-LD on *A*. *stephensi* competence. (A) PGRP-LD protects gut microbiota by negatively regulating immune responses. The gut microbes promote integrity of PM, which enhances vector resistance to parasite infection. (B) Knock down of *pgrp-ld* leads to the upregulation of immune effectors, which kills a significant number of gut microbes. Dysbiosis of the gut microbiota results in the presentation of a structurally compromised PM, which facilitates *P*. *berghei* infection.

In both invertebrates and vertebrates PGRPs play important roles in regulating interactions with pathogens and commensal bacteria [9]. In this study, we show that PGRP-LD protects *A*. *stephensi* from parasite infection by regulating homeostasis of the mosquito’s gut microbiota (Fig 7). Reduced *pgrp-ld* activates the host immune system, which depletes the abundance of gut microbes in this niche. This impairs PM structure and increases susceptibility to parasite infection.

PGRP family members were first identified because they share a conserved PGRP domain that is able to detect peptidoglycan (PGN) present on the cell wall of both Gram^+^ and Gram^-^ bacteria [9,33]. Recent studies using disease vectors show that PGRPs also play important roles in parasite defense [9,12,34–36]. The function of PGRP-LC is well characterized in *Anopheles* mosquitoes and the tsetse fly, where the protein is responsible for initiating synthesis of downstream effectors in response to both native microbes and invading pathogens [9,11,37]. PGRP-LA participates in defense against parasite infection by functioning similarly to PGRP-LC [12]. PGRP-LB acts as a negative regulator of Imd signaling pathway through its amidase activity [12,38–40]. In tsetse, PGRP-LB has evolved to exhibit bactericidal and anti-parasitic activity [41]. Unlike the above-mentioned PGRPs, little is known about the mechanistic role of PGRP-LD in pathogen defense in either *Drosophila* or other insects, except that it protects *Armigeres* mosquitoes from *E*.*coli* infection by modulating expression of downstream antimicrobial peptides [42]. We show here that *A*. *stephesi* PGRP-LD promotes host defense against *P*. *berghei*. Experimental knock down of *pgrp-ld* expression induces the expression of downstream effectors both in the presence or absence of parasite challenge. Based on its structure, *A*. *stephensi* PGRP-LD lacks conserved residues essential for either PGN binding or amidase activity, which has been identified in *Drosophila* PGRPs [43–45]. This is in contrast with most of PGRPs, which function as negative regulators that prevent over activation of immune signaling pathways by catabolizing immunostimulatory peptidoglycan [46,47]. One explanation is that PGRP-LD may use less well conserved residues to bind peptidoglycan. Alternatively, PGRP-LD may interfere the signal transduction of immune pathways, as does *Drosophila* PGRP-LF that dampens Imd signaling strength by interfering with PGRP-LC-peptidoglycan binding activity [48]. Further investigations are required to determine how PGRP-LD regulates immune system function.

The gut microbiota enhances host intestinal barrier function and pathogen tolerance in both vertebrates and invertebrates [47,49]. In *A*. *stephensi*, *pgrp-ld* knockdown elevates immune activity that eliminates the majority of gut microbes but fails to eliminate *P*. *berghei*. In these mosquitoes, the spatial structure of remaining bacteria was altered. Gut microbes that are usually restrained within the endoperitrophic space localize in close contact with midgut epithelium. Our results indicate that PM structure is compromised in dsLD treated mosquitoes. We then observe PM structure is impaired and expression of PM genes varies significantly. These results suggest that the defect of PM structure results from the dysregulation of PM genes. In addition, we also find that the PM of *A*. *stephensi* is absent 48 hr post blood meal in antibiotic treated mosquitoes in which most enteric microbes are cleared. This defect is also associated with decreasing *peritrophin* and increasing *chitinase* expression. Thus PM structural integrity is associated with the homeostasis of gut bacteria *in A*. *stephensi*, similarly as in many disease vectors [27–29]. When mosquitoes treated with antibiotics are re-colonized by *Enterobacter sp*, both PM structural integrity and vector competence are restored. In agreement with the finding in *An*. *coluzzii* that PM is induced by gut microbiota [29], our results further confirm that gut microbiota of *Anopheles* mosquitoes is essential for PM integrity. However, we are currently unable to say at what abundance and how gut microbiota are able to maintain intact PM structure.

The PM serves as a physical barrier to parasite infection establishment in multiple disease transmitting vectors, including tsetse flies, sand flies and ticks [27,32,50–56]. Our study shows that in antibiotic treated mosquitoes that present a compromised PM, knockdown of *pgrp-ld* expression does not change infection prevalence compared to controls. This result reinforces that increasing susceptibility of dsLD mosquitoes to *P*. *berghei* infection is due to the comprised PM as opposed to reduced levels as of PGRP-LD directly. In agreement with most vectors, our results show that PM is a major physical barrier that prevents *P*. *berghei* infection establishment in *A*. *stephensi*.

In summary, our data demonstrate that a complex interplay exists between the host immune system, gut microbes and the PM, and this interplay determines parasite infection outcomes in *A*. *stephensi*. PGRP-LD, functioning as a key mediator, helps to maintain this balance. Detailed studies on the regulation of PGRP-LD on immune signaling pathways, and the influence of gut microbiota on PM formation, are currently under way and may provide new insights into interactions between immune system, gut microbiota and parasites.

## Materials and methods

### Ethics statement

All animals were handled according to the guidelines for the Care and Use of Laboratory Animals of the National Institutes of Health and the Office of Laboratory Animal Welfare. The research protocol was approved by the Institutional animal care and use committee, Department of Laboratory Animal Science, Fudan University (IACUC 20161784A359).

### Mosquito rearing and antibiotic treatment

The *Anopheles stephensi* mosquito (strain Hor) was reared at 28°C, 80% relative humidity and at a 12h light/dark cycle. Adults were maintained on 10% sucrose and BALB/c mice. Newly eclosed mosquitoes were administrated with fresh filtered 10% sucrose supplemented with 10 U/ml penicillin, 10 μg/ml streptomycin and 15 μg/ml gentamicin daily, for up to 5 days [13].

### Gene silencing and reverse transcription quantitative PCR (RT-qPCR)

PCR amplicons tailed with T7 promoter sequences were used to synthesize dsRNAs using MEGAscript RNA kit (Ambion, Invitrogen). The cDNA clones *Astepgrp-ld* (ASTE010245), and plasmid eGFP (BD Biosciences) served as templates for amplification using gene specific primers (S1 Table). Five to 6-day-old females received a total 69 nl dsRNAs (4μg/μl) injected intra-thoracically using nanoject II microinjector (Drummond). Injected mosquitoes were allowed to recover for 5 days prior to infection [57]. Survival rate was recorded daily for 5 days post dsRNA treatments and compared to that of dsGFP controls. Silencing efficiency was verified by qPCR 2-day post dsRNA treatment with primers listed in S1 Table. RNA was extracted from flash frozen mosquitoes utilizing the standard TRI reagent (Sigma-Aldrich, China) protocol. cDNA was prepared from total RNA using the 5XAll-in-One MasterMix (with AccuRT Genomic DNA Removal Kit) (ABM, China). Levels of target genes were determined by Roche LightCycler 96 Real Time PCR Detection System with SYBR Green qPCR Master Mix (Biomake, China) using the following conditions: 95°C for 5 min, 40 cycles of 15 sec at 95°C, 30 sec at 60°C, and 15 sec at 72°C. Fluorescence readings were taken at 72°C after each cycle. Melting curves (60°C–95°C) were performed to confirm the identity of the PCR product. The data were processed and analyzed with LightCycler 96 software. Expression of *cecropin*, *gambicin*, *defensin*, *tep1*, *prophenoloxidase*, *nos*, *duox* and *caudal* were analyzed 5 days post dsRNA administration with primers listed in S1 Table. Ribosomal gene *S7* widely used in studies of *Anopheles* gene expression was used as the internal reference [58–61]. PCR efficiency of each primer set was determined by standard curve. Relative quantitation results were normalized with *S7* and analyzed by the 2^–ΔΔCt^ method [62]. Gene expression of dsLD treated group was normalized to dsGFP controls. The normality of data sets was determined by Shapiro-Wilk test before t test analysis. Values are represented as the mean (±SEM), and statistical significance was determined using a Student’s t test and Excel software.

### *Plasmodium* infection

*A*. *stephensi* were starved overnight and then fed on *P*. *berghei* (ANKA) infected BALB/c with parasitemia of 6–7% using standard protocols [63]. Mosquitoes were starved for 24 hr before blood feeding. After imbibing a blood meal, mosquitoes were maintained at 21°C. Un-engorged mosquitoes were removed 24hr post blood meal. Midguts were dissected and infection intensity were determined microscopically 8-day post infection. The oocyst data were not normally distributed as determined by Shapiro-Wilk test. Thus, significance was determined using the Mann-Whitney test.

### Microbiota analysis

Mosquitoes were collected 5 day after dsRNA treatment or antibiotic treatment and surface sterilized with 70% ethanol twice and 0.9% NaCl twice. Midguts were dissected and homogenized in 0.9% NaCl. Homogenates were serially diluted and plated on LB agar plates. CFUs were counted 2 days after incubation at 28°C. Total DNA was extracted by the method of Holmes and Bonner as described [64]. Bacterial density was quantified by qPCR using universal 16S rRNA primers [28] (S1 Table). Ribosomal gene S7 was used as the internal reference. Significance was determined using the Student’s t-test.

The composition of the gut microbiota in dsRNA treated mosquitoes was analyzed by pyrosequencing that targeted the V3-V4 region of bacterial 16S rRNA [65]. 10 midguts of dsRNA treated mosquitoes were pooled for 1 biological replicate. DNA of 3 biological replicates of each treatment were prepared for further sequencing analysis (S1 Text).

For fluorescent in situ hybridization (FISH), abdomens of dsLD treated females 2 day post blood meal were fixed and sectioned as described [66]. Slides were hybridized with 10ng/μl universal 16S ribosomal RNA probe (5’-GCTGCCTCCCGTAGGAGT-3’) labeled with Alexa Fluor 555 (Life technology). Tissues were visualized using Nikon ECLIPSE IVi microscope connected to a Nikon DIGITAL SIGHT DS-U3 digital camera.

### Peritrophic matrix analysis

Forty-eight hour post blood meal mosquito abdomens were fixed and sectioned as described above [66]. Samples were sectioned at 5 μm, stained with hematoxylin and eosin (H&E) (Huntz Enterprises Inc., China) and Periodic Acid Schiff (PAS) (Sigma-Aldrich, China) according to the manufacturer’s protocol. Slides were hard mounted using Canada balsam (ChemsWorth). Slides were viewed using bright field illumination on a Nikon ECLIPSE IVi microscope connected to a Nikon DIGITAL SIGHT DS-U3 digital camera. Four days post dsRNA treatment *A*. *stephensi* were fed with blood meal supplemented with 500 kDa FITC-labeled dextran molecules (2.5mg/ml blood)(Sigma) which were filtered using PD MiniTrap Sephadex G10 columns (GE Healthcare) as described [27]. Forty eight hours post-feeding, midguts were dissected and FITC signal observed using a Zeiss, LSM710 confocal microscope connected to a Nikon DIGITAL SIGHT DS-U3 digital camera. Expression of 4 PM genes was analyzed 24 hr and 48 hr post blood meal using primers targeting *peritrophin1*(ASTE010406), *peritrophin14* (ASTE009456), 2 *chitinases*, herein named *chitinaseA* (ASTE005630) and *chitinaseB* (ASTE000328) (S1 Table).

### Oral administration of bacteria

The administration of mosquito commensal bacteria was performed as described [67]. Briefly, an overnight culture of *Enterobacter sp*. was washed 2 times in phosphate-buffered saline (PBS) and introduced to mosquitoes via a sugar meal. A final concentration of 1X10^5^~1X10^7^ /ml bacteria was added to 1.5% sterile sugar. All mosquitoes were starved for 24hr hours before being offered a blood meal. Mosquitoes were given a blood meal 2-day post bacteria treatment. Age matched wild-type and antibiotic treated mosquitoes were used as controls.

## Supporting information

S1 Text Materials and methods. (DOCX)

S1 Table Primers used for gene cloning, dsRNA preparation, 16S rRNA pyrosequencing and quantitative PCR. (DOCX)

S1 Fig Alignment of conserved PGRP domains from *A*. *stephensi* and other insect vectors. PGRPs from *Anopheles stephensi*: AstePGRP-LD (ASTE010245), AstePGRP-LB (ASTE006009); *Anopheles gambiae*: AgPGRP-LC (AGAP005203), AgPGRP-LD (AGAP005552), AgGPRP-LB (AGAP001212); *Drosophila melanogaster*: DmPGRP-LCx (FBGN0035976), DmPGRP-LB (FBGN0037906), DmPGRP-LD (FBGN0260458), DmPGRP-SA (FBGN0030310) and *Glossina morsitans morsitans*: GmmPGRP-LC (GMOY006094), GmmPGRP-LD (GMOY004195), GmmPGRP-LB (GMOY006730). Three conserved PGRP domains are boxed in black and numbered. The highly conserved residues among all PGRP proteins are shown in grey, conserved residues present in the recognition PGRPs and catalytic PGRPs are shown in light grey shadow. Residues required for amidase activity are indicated by a star at the bottom. Residues required for peptidoglycan binding in DmPGRP-LCx, DmPGRP-SA are indicated by diamond and triangle, respectively.
(TIF)

S2 Fig Survival curve of dsRNA treated *A*. *stephensi*. Survival was recorded daily for 5 days post dsRNA treatments and compared to that of dsGFP controls. No significant difference was seen between dsLD and dsGFP mosquitoes. The data are the representative of three replicate infections. Total sample size: dsGFP (n = 30), dsLD (n = 30).
(TIF)

S3 Fig Population community of dsRNA treated mosquitoes by 16s rRNA pyrosequencing. (A) Incidence of the major bacterial taxonomic in dsGFP and dsLD. Relative abundance of identified microbial taxa in the midguts collected from mosquitoes 5 day post dsRNA treatment. (B) Principal coordinates analysis of the bacterial composition in dsGFP (red dots) and dsLD (green dots) at operational taxonomic unit (OTU) (97%) level. Each sample contains 10 midguts.
(TIF)

S4 Fig Analysis of PM structure by PAS staining. (A) PM structure was observed in dsGFP (A1) and dsLD (A2) mosquitoes at 200X magnification. (B) PM structure was observed in normal (B1), antibiotic treated mosquitoes (B2) and antibiotic treated mosquitoes recolonized with 1X10^5^/ml *Enterobacter sp*. (B3) at 200X magnification. Arrows denote the PM. Images are representative of three independent experiments. Scale bars, 50 μm.
(TIF)

S5 Fig Bacterial abundance of culturable (A) and total gut microbiota (B) in normal and antibiotics treated mosquitoes (n = 10). Error bars indicate standard error. Images are representative of three independent experiments.
(TIF)
