# Supplementary material for: PGRP-LD mediates A. stephensi vector competency by regulating homeostasis of microbiota-induced peritrophic matrix synthesis
Source: PLoS Pathog. 2018 Feb 28;14(2):e1006899. doi: 10.1371/journal.ppat.1006899 (PMC5831637; doi:10.1371/journal.ppat.1006899)
Supplement: S1 Text — (DOCX) [file ppat.1006899.s001.docx]

**Materials and Methods**

Bioinformatics analysis of the 16S rRNA gene sequencing data

The composition of gut microbiota in dsRNA treated mosquitoes were analyzed by pyrosequencing targeting V3-V4 region of bacterial 16S rRNA using primer sets (341F, 806R) [1]. Amplification, barcoding and sequencing were completed by Novogen, China, using the HiSeq250 platform. Raw reads were filtered and assembled by QIIME (Version 1.7.0) [2] and FLASH [3] software packages. Operational Taxonomic Units (OTU), defined by a similarity of 97%, were picked using the UCLUST method[2]. Taxonomy assignment was obtained by RDP Classifier [4]. Representative sequence for each OTU was screened for further annotation. For each representative sequence, the GreenGene Database (http://greengenes.lbl.gov/cgi-bin/nph-index.cgi) [5] was used based on RDP. Beta diversity were evaluated to compare dsGFP and dsLD treated groups through QIIME. To visualize the variation between groups, PCA plots were generated using the FactoMineR package and ggplot2 package in R software(Version 2.15.3). The 16S rRNA gene sequences are available at the National Center for Biotechnology Information’s Sequence Read Archive (accession no. SRP102164).

**References**

1. Michelsen CF PP, Glaring MA, Schjoerring JK, Stougaard P (2014) Bacterial diversity in Greenlandic soils as affected by potato cropping and inorganic versus organic fertilization. Polar Biology 37: 61-71.

2. Caporaso JG, Kuczynski J, Stombaugh J, Bittinger K, Bushman FD, et al. (2010) QIIME allows analysis of high-throughput community sequencing data. Nat Methods 7: 335-336.

3. Magoc T, Salzberg SL (2011) FLASH: fast length adjustment of short reads to improve genome assemblies. Bioinformatics 27: 2957-2963.

4. Wang Q, Garrity GM, Tiedje JM, Cole JR (2007) Naive Bayesian classifier for rapid assignment of rRNA sequences into the new bacterial taxonomy. Appl Environ Microbiol 73: 5261-5267.

5. DeSantis TZ, Hugenholtz P, Larsen N, Rojas M, Brodie EL, et al. (2006) Greengenes, a chimera-checked 16S rRNA gene database and workbench compatible with ARB. Appl Environ Microbiol 72: 5069-5072.
