# Supplementary material for: PGRP-LD mediates A. stephensi vector competency by regulating homeostasis of microbiota-induced peritrophic matrix synthesis
Source: PLoS Pathog. 2018 Feb 28;14(2):e1006899. doi: 10.1371/journal.ppat.1006899 (PMC5831637; doi:10.1371/journal.ppat.1006899)
Supplement: S1 Table — (DOCX) [file ppat.1006899.s002.docx]

| Primer name  (accession #) | Primer sequences  (F, Forward; R, reverse) | Amplicon size  (bp) | Tm  (℃) |
| --- | --- | --- | --- |
| *PGRP-LD  (ASTE010245) | F: 5' GTTAATCGTACTGAACAG 3' | 401 | 50 |
|  | R: 5' CAAGCAACAGGAATATCA 3' |  |  |
| ^∆^T7-PGRP-LD | F: 5' TAATACGACTCACTATAGGGTTAATCGTACTGAACAG 3' | 401 | 60 |
|  | R: 5' TAATACGACTCACTATAGGCAAGCAACAGGAATATCA 3' |  |  |
| ^∆^T7-GFP | F: 5' TAATACGACTCACTATAGGGTCAGTGGAGAGGGTGAAG 3' | 454 | 60 |
| (BD Biosciences) | R: 5' TAATACGACTCACTATAGGCTAGTTGAACGGATCCATC 3' |  |  |
| qPGRP-LD | F: 5' GCCCGTTTCGCATCCGTTCT 3' | 126 | 60 |
|  | R: 5' TCCGCACCCGTATCCACCAC 3' |  |  |
| qS7  （ASTE004816） | F: 5' TGCGGAGCGTCGTATTCTGC 3' | 79 | 60 |
|  | R: 5' ACACAGCGGTGAGCGTTCG 3' |  |  |
| qCEC  （ASTE007106） | F: 5' GCTGCTCTTTCTCGTTGCG 3' | 98 | 60 |
|  | R: 5' CGGCACCTTCCACCTTCT 3' |  |  |
| qGAM  （ASTE002252） | F: 5' CCGCTGTTCGTCCTCGTTCA 3' | 91 | 60 |
|  | R: 5' GCACACGGACGCCAACTTCT 3' |  |  |
| qDEF  （ASTE011281） | F: 5' AGTCGTGGTCCTGGCGGCTCT 3' | 208 | [1] |
|  | R: 5' ACGAGCGATGCAATGCGCGGCA 3' |  |  |
| qTEP1  （ASTE010227） | F: 5' CCTGGGTGCGTGGGAAAC 3' | 106 | 60 |
|  | R: 5' GCCTTGCTGTCGTTCGTGAT 3' |  |  |
| qPPO  （ASTE000587） | F: 5' TTCTGCGGTTGCGGCTGG 3' | 106 | 60 |
|  | R: 5' CGGCGTCTTGCTCGTAGTCG 3' |  |  |
| qNOS  （ASTE008593） | F: 5' CAGCGAACGGACGGCAAGCA 3' | 186 | 60 |
|  | R: 5' TGACACGACCAGCGGCAGGAT 3' |  |  |
| qDUOX  （ASTE003295） | F: 5' TCGTGAGCGTCGTCAGAAGC 3' | 116 | 60 |
|  | R: 5' CCTCACCGTCCAGCGATGC 3' |  |  |
| q16s | F: 5' AGAGTTTGATCCTGGCTCAG 3' | 250 | [2] |
|  | R: 5' CATGCTGCCTCCCGTAGGAGT 3' |  |  |
| V3-V4 | F: 5' CCTAYGGGRBGCASCAG 3' | 249 | [3] |
|  | R: 5' GGACTACNNGGGTATCTAAT 3' |  |  |
| qPer1 | F: 5' AGCCACGGCAATGCGGTTGT3' | 140 | 60 |
| （ASTE010406） | R: 5' TCGGACGGTTGCAGCGGTTG3' |  |  |
| qPer14 | F: 5' TGGTGCTCGCTACGTTCGCT3' | 172 | 60 |
| （ASTE009456） | R: 5' TGCAAACCGCCGGGACAGT3' |  |  |
| qChitinaseA | F: 5' CGTCTGCTGCTGACTGCTGCC3' | 141 | 60 |
| （ASTE005630） | R: 5' TCCAACCTGCCGTTCCCACTGT3' |  |  |
| qChitinaseB | F: 5' AACGGGCTCGGTGGTATCAT3' | 103 | 60 |
| （ASTE000328） | R: 5' GTGCTTCCTTGGCTGCTTCA3' |  |  |
| qPGRP-LC | F:5' TGTGCCATCGTAGCGGTCAT3' | 98 | 60 |
| （ASTE002618） | R:5' AGCCACTCGGTTCTCGTCAC3' |  |  |
| qPGRP-LB | F: 5' GGCGATTGGGTTGCGGATTT3' | 116 | 60 |
| （ASTE006009） | R: 5' CGATGTCCGAGCAGGGTGTA3' |  |  |
| qPGRP-LA | F:5' GCGGCGACAGACCAAACC3' | 110 | 60 |
| （ASTE002619） | R:5' CACAGTGGGCGTATGCTTGC3' |  |  |
| qCAU | F: 5'TGGCGGTCTCGGCGGTCAA3'  R: 5'GGTGGCTGGGATGGTGGTGGT3' | 200 | 60 |
| (ASTE016107) |  |  |  |

*: Primers for gene cloning

^∆^: Primers for dsRNA synthesis

q: Primers for real time quantitative PCR

The references of cited primers were listed in Tm column:

[1] Joshi D, Pan X, McFadden MJ, Bevins D, Liang X, et al. (2017) The Maternally Inheritable *Wolbachia* *wAlbB* Induces Refractoriness to *Plasmodium berghei* in *Anopheles stephensi*. Front Microbiol 8: 366.

[2] Narasimhan S, Rajeevan N, Liu L, Zhao YO, Heisig J, et al. (2014) Gut microbiota of the tick vector *Ixodes scapularis* modulate colonization of the Lyme disease spirochete. Cell Host Microbe 15: 58-71.

[3] Michelsen CF PP, Glaring MA, Schjoerring JK, Stougaard P (2014) Bacterial diversity in Greenlandic soils as affected by potato cropping and inorganic versus organic fertilization. Polar Biology 37: 61-71.
