# Supplementary figures and images for: PGRP-LD mediates A. stephensi vector competency by regulating homeostasis of microbiota-induced peritrophic matrix synthesis
Source: PLoS Pathog. 2018 Feb 28;14(2):e1006899. doi: 10.1371/journal.ppat.1006899 (PMC5831637; doi:10.1371/journal.ppat.1006899)

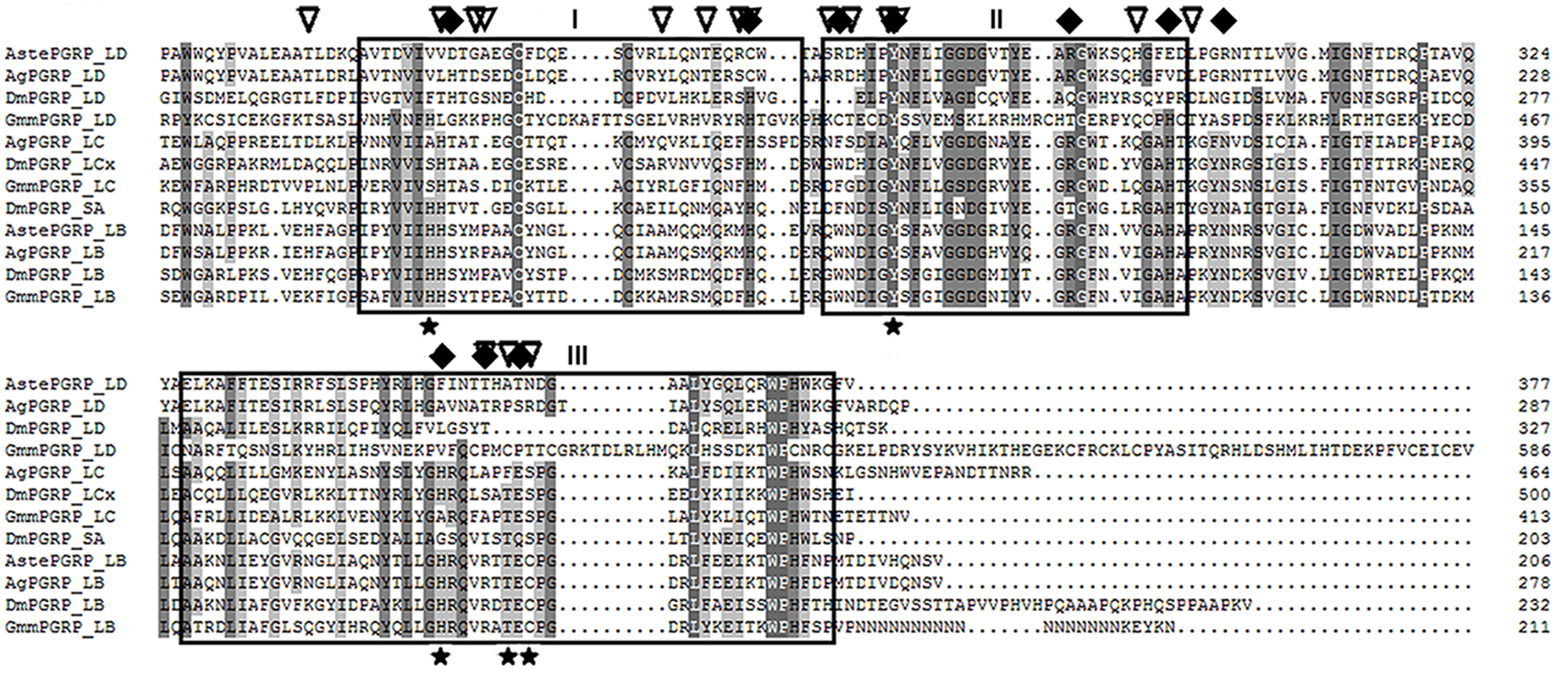

Supplement: S1 Fig — PGRPs from Anopheles stephensi: AstePGRP-LD (ASTE010245), AstePGRP-LB (ASTE006009); Anopheles gambiae: AgPGRP-LC (AGAP005203), AgPGRP-LD (AGAP005552), AgGPRP-LB (AGAP001212); Drosophila melanogaster: DmPGRP-LCx (FBGN0035976), DmPGRP-LB (FBGN0037906), DmPGRP-LD (FBGN0260458), DmPGRP-SA (FBGN0030310) and Glossina morsitans morsitans: GmmPGRP-LC (GMOY006094), GmmPGRP-LD (GMOY004195), GmmPGRP-LB (GMOY006730). Three conserved PGRP domains are boxed in black and numbered. The highly conserved residues among all PGRP proteins are shown in grey, conserved residues present in the recognition PGRPs and catalytic PGRPs are shown in light grey shadow. Residues required for amidase activity are indicated by a star at the bottom. Residues required for peptidoglycan binding in DmPGRP-LCx, DmPGRP-SA are indicated by diamond and triangle, respectively. (TIF) [file ppat.1006899.s003.tif]

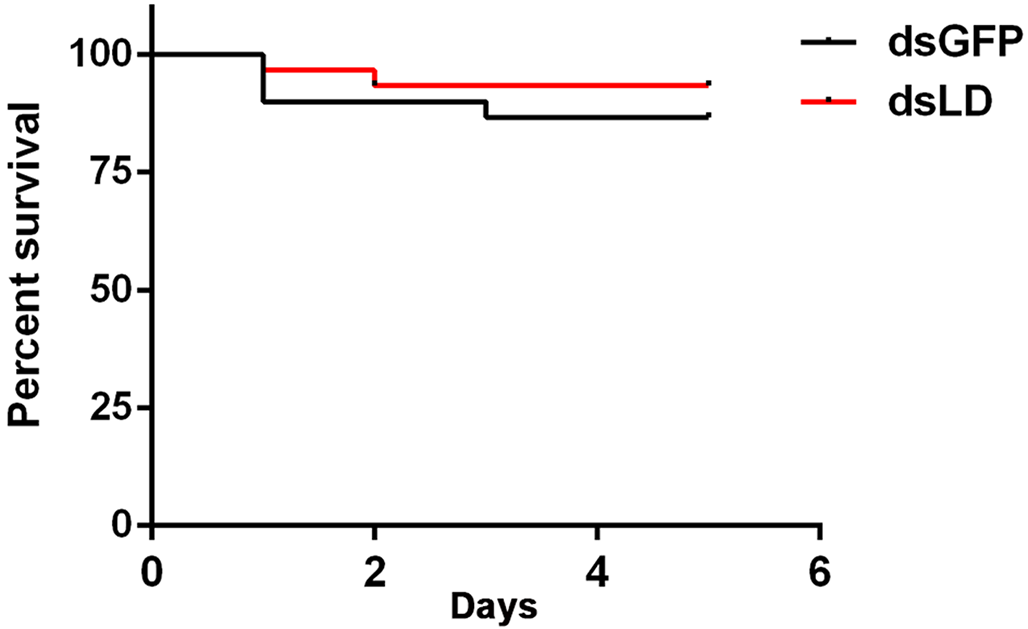

Supplement: S2 Fig — Survival was recorded daily for 5 days post dsRNA treatments and compared to that of dsGFP controls. No significant difference was seen between dsLD and dsGFP mosquitoes. The data are the representative of three replicate infections. Total sample size: dsGFP (n = 30), dsLD (n = 30). (TIF) [file ppat.1006899.s004.tif]

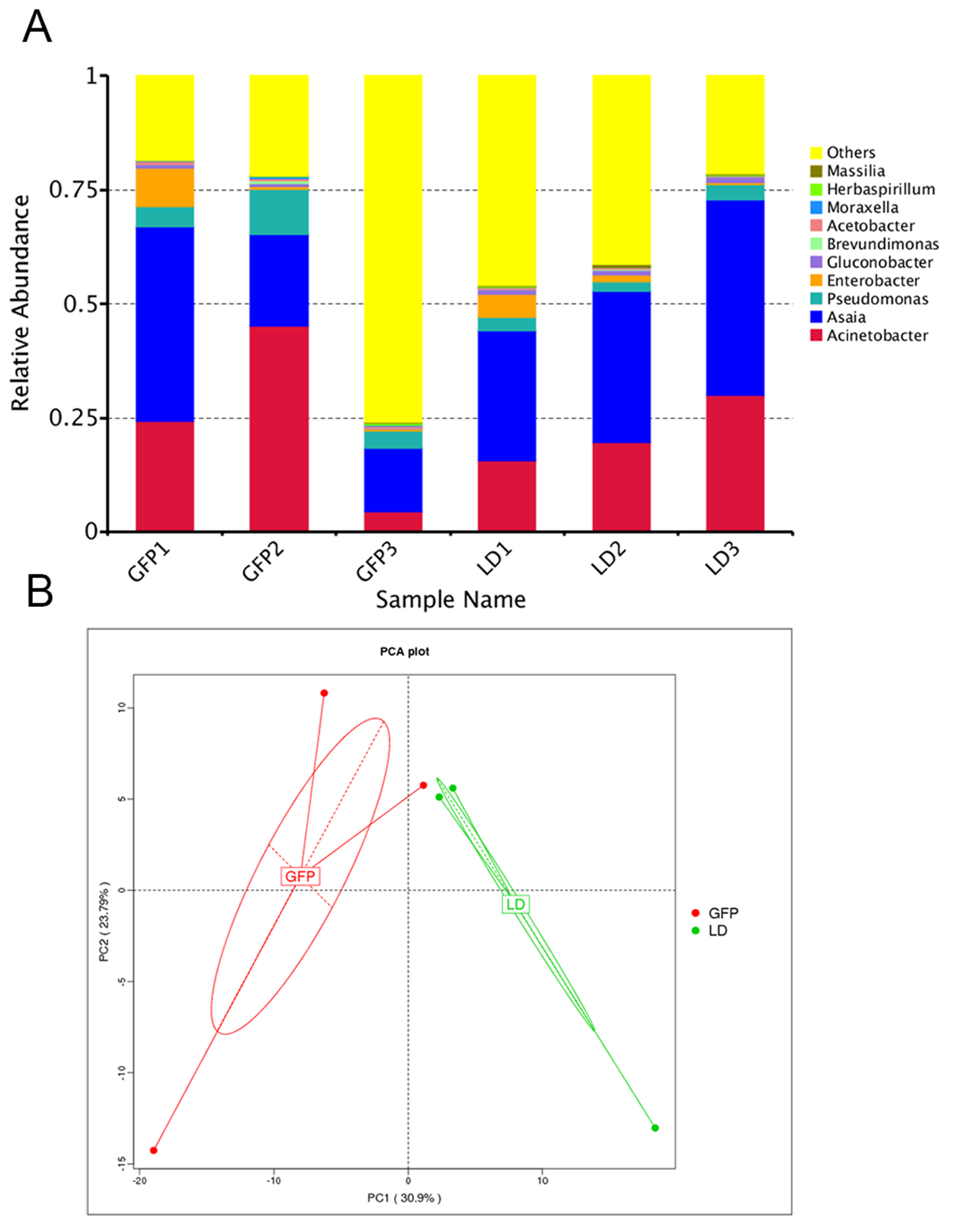

Supplement: S3 Fig — (A) Incidence of the major bacterial taxonomic in dsGFP and dsLD. Relative abundance of identified microbial taxa in the midguts collected from mosquitoes 5 day post dsRNA treatment. (B) Principal coordinates analysis of the bacterial composition in dsGFP (red dots) and dsLD (green dots) at operational taxonomic unit (OTU) (97%) level. Each sample contains 10 midguts. (TIF) [file ppat.1006899.s005.tif]

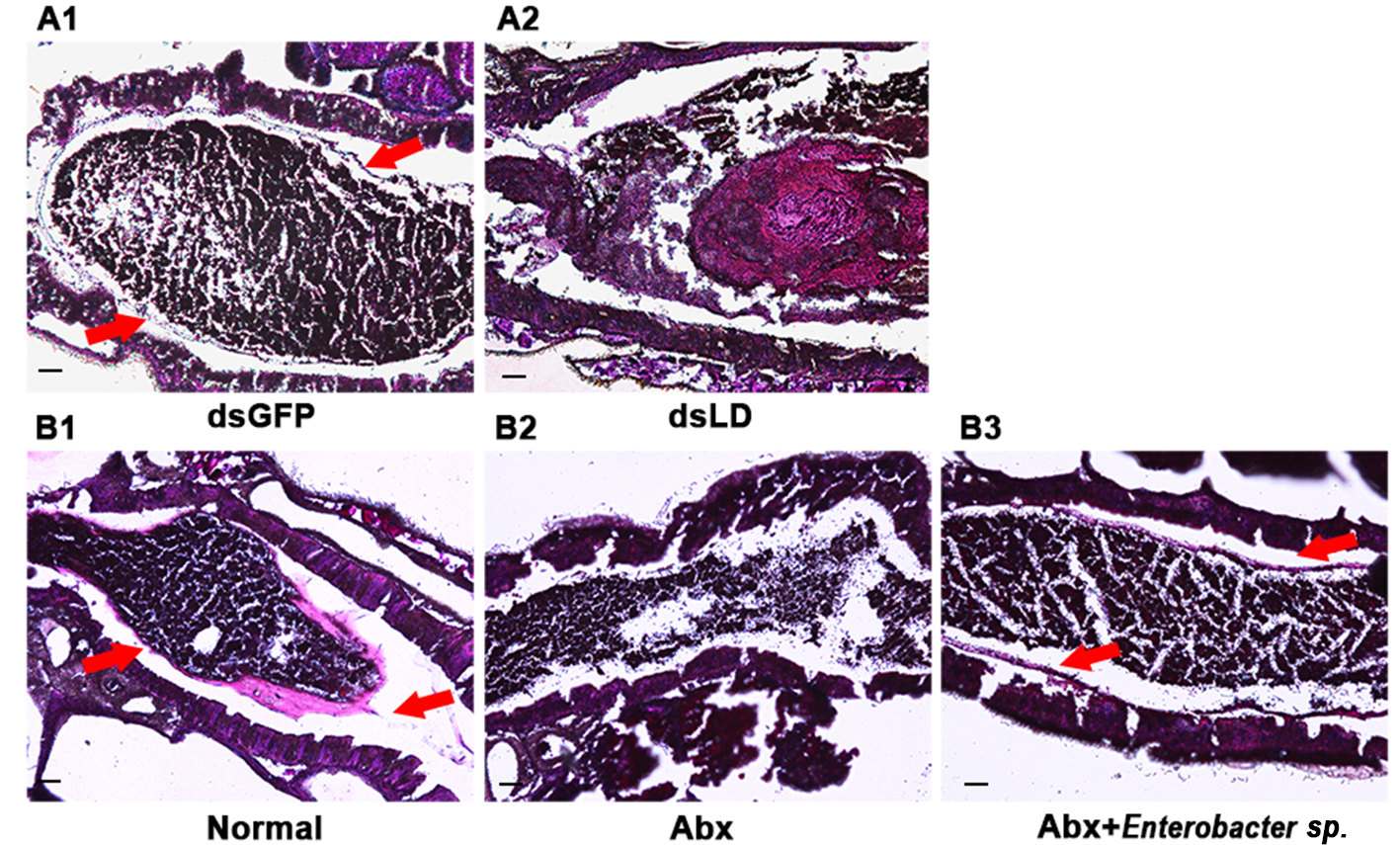

Supplement: S4 Fig — (A) PM structure was observed in dsGFP (A1) and dsLD (A2) mosquitoes at 200X magnification. (B) PM structure was observed in normal (B1), antibiotic treated mosquitoes (B2) and antibiotic treated mosquitoes recolonized with 1X105/ml Enterobacter sp. (B3) at 200X magnification. Arrows denote the PM. Images are representative of three independent experiments. Scale bars, 50 μm. (TIF) [file ppat.1006899.s006.tif]

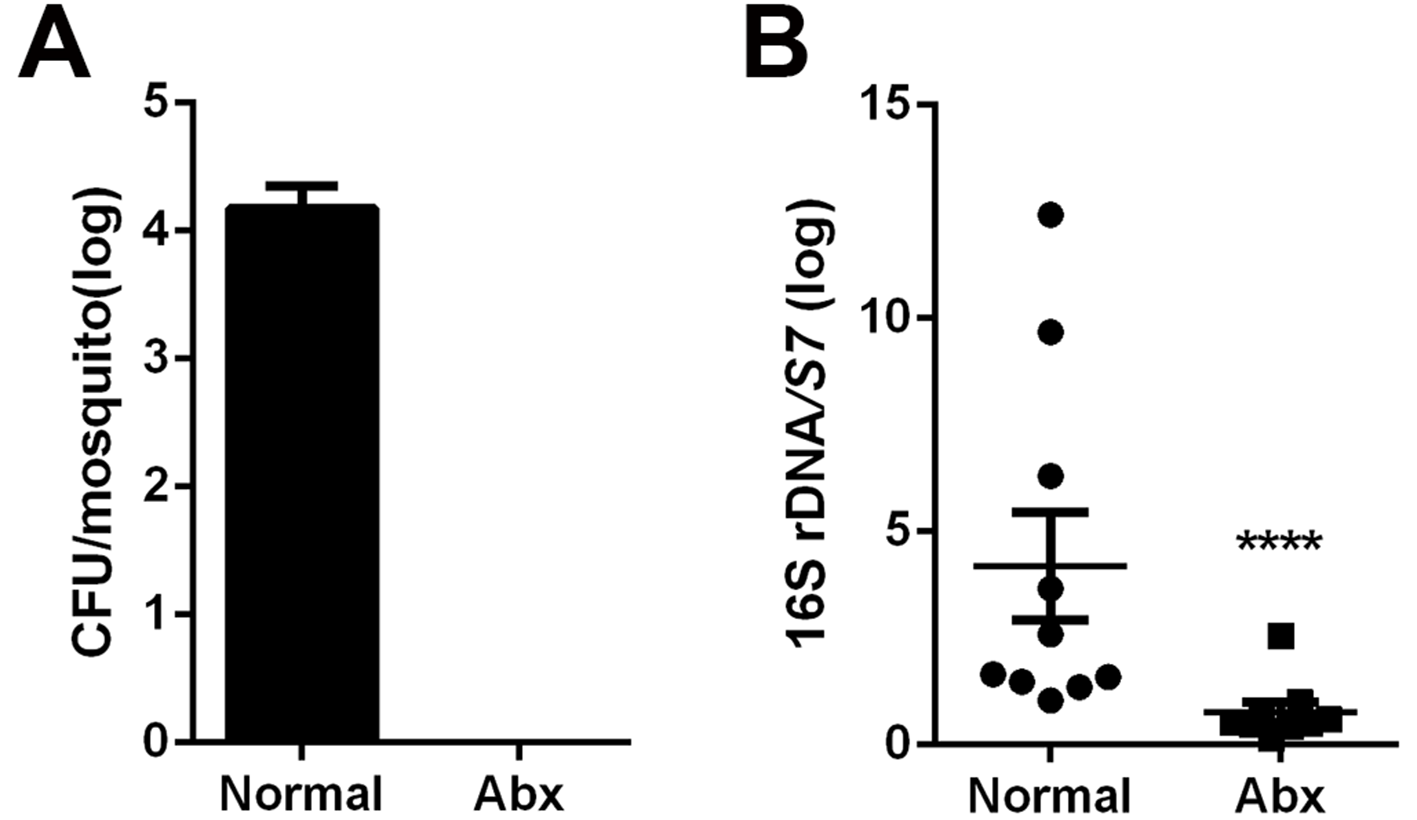

Supplement: S5 Fig — Error bars indicate standard error. Images are representative of three independent experiments. (TIF) [file ppat.1006899.s007.tif]
